# β-receptor blocker enhances anti-tumor immunity via inhibiting lactate-induced norepinephrine metabolism of macrophages during malignant pleural effusion

**DOI:** 10.3389/fimmu.2024.1497468

**Published:** 2025-01-03

**Authors:** Ru Zhang, Weijia Wang, Aitian Li, Huishang Wang, Xiaoyan Liu, Feifei Fan, Ying Wang, Huanyu Zhang, Jingxia Chang, Yinyin Zhang, Hongmin Wang, Lijun Miao, Bo Huang, Li Yang, Yi Zhang

**Affiliations:** ^1^ Biotherapy Center and Cancer Center, The First Affiliated Hospital of Zhengzhou University, Zhengzhou, Henan, China; ^2^ Respiratory Department, The First Affiliated Hospital of Zhengzhou University, Zhengzhou, Henan, China; ^3^ Hematology Department, The First Affiliated Hospital of Zhengzhou University, Zhengzhou, Henan, China; ^4^ Department of Immunology and National Key Laboratory of Medical Molecular Biology, Institute of Basic Medical Sciences, Chinese Academy of Medical Sciences, Beijing, China; ^5^ State Key Laboratory of Esophageal Cancer Prevention & Treatment, Zhengzhou, Henan, China; ^6^ School of Life Sciences, Zhengzhou University, Zhengzhou, Henan, China; ^7^ Tianjian Laboratory of Advanced Biomedical Sciences, Academy of Medical Sciences, Zhengzhou University, Zhengzhou, Henan, China; ^8^ School of Public Health, Zhengzhou University, Zhengzhou, Henan, China

**Keywords:** macrophage, tumor immunity, norepinephrine metabolism, lactate, β-receptor blocker

## Abstract

**Introduction:**

Malignant pleural effusion (MPE) is associated with poor quality of life and mortality in patients with tumors. In clinical practice, we observed that patients with malignant pleural effusion (MPE) and concurrent heart disease exhibited a decrease in MPE volumes following treatment with β-receptor blockers for heart disease. Immunosuppressive tumor microenvironment was found to play a substantial role in the progression of MPE, and mainly attributed to tumor-associated macrophages (TAMs). However, whether β-receptor blockers improve MPE through affecting the immune microenvironment especially TAMs and the potential mechanism behind remains unclear.

**Methods:**

In this study, we collected the MPE samples from MPE and heart disease patients treated with propranolol, and performed flow cytometry experiment to evaluate the effect of propranolol on MPE immune microenvironment. Then, the mechanism that how propranolol effectively reprogrammed the immunosuppressive microenvironment of MPE was conducted by the experiments of mass spectrometry, RNA-seq, flow cytometry, immunofluorescence, western blotting, etc. Lastly, to further evaluate the effect of propranolol on MPE therapy in vivo, we developed a mouse model of MPE. We administrated propranolol into MPE-bearing mice to investigate the therapy efficacy and the changes of MPE microenvironment by the experiments of computed tomography (CT) scanning, flow cytometry, etc.

**Results:**

We observed that propranolol treatment in MPE patients with heart disease decreased TAM frequency and immunosuppression and enhanced anti-tumor immunity. Macrophages in MPE exhibited an immunosuppressive phenotype via the activation of norepinephrine metabolism. Subsequently, we found that lactate was increased in MPE and may contribute to an increase in TAM frequency and inhibition of anti-tumor immunity by macrophages. Additionally, lactate triggered phenylalanine/norepinephrine signaling and further induced macrophage immunosuppression in an ERK-depended way. Lastly, in the MPE mouse model, propranolol inhibited MPE development and reversed the immune microenvironment of MPE.

**Discussion:**

Here, we reveal the mechanism by which lactate induces macrophage immunosuppression via activating phenylalanine/norepinephrine signaling. Our findings highlight that blocking norepinephrine signaling by β-receptor blockers is an attractive therapeutic strategy to enhance anti-tumor immunity in the context of MPE

## Introduction

Malignant pleural effusion (MPE) is a common condition that correlates with poor quality of life and mortality in patients with cancer (1). MPE is a manifestation of advanced cancer (2), and develops an immune microenvironment during effusions formation (3). The immune microenvironment plays a pivotal role in the progression of MPE (3). In clinic, we previously observed that patients had MPE and combined with heart disease showed a reduction in MPE volumes after receiving the β-receptor blocker for heart disease therapy. β-receptor blockers are primarily used to treat cardiovascular diseases such as hypertension, heart failure, and arrhythmias (4, 5). Previous studies have shown that the use of β-receptor blockers improves survival in some cancers (6, 7). It was suggested that β-receptor blockers may improve MPE by potentially affecting the immune microenvironment.

In our previous study, we demonstrated that tumor-associated macrophages (TAMs) can serve as biomarkers for the diagnosis, prognosis, and therapeutic response of MPE (8, 9). Furthermore, TAMs play a substantial role in the immunosuppressive tumor microenvironment of MPE (10). TAMs are an essential proportion of tumor-infiltrating immune cells, and their high plasticity and corresponding phenotypic polarization significantly contribute to tumor progression. Thus, we hypothesized whether β-receptor blocker affected the immunosuppressive function of TAMs to improve MPE.

β-receptor blockers suppress cellular norepinephrine-related signaling and impact cell function (11). Norepinephrine is synthesized from L-tyrosine via a series of enzymatic steps involving tyrosine hydroxylase, aromatic L-amino acid decarboxylase, and dopamine β-hydroxylase (12). Recent studies have indicated that norepinephrine could influence the adrenergic receptors of myeloid-derived suppressor cells, thereby regulating their expression of immunosuppressive molecules such as arginase-1 and PD-L1, and increasing their capacity to inhibit the proliferation of T cells (13). In another study, it was reported that norepinephrine can inhibit the proliferation of T cells and the production of cytokines (11).

Increasing evidence strongly links cellular metabolism to immune cell effector function and exhaustion (14–16). Influenced by metabolic reprogramming within the tumor microenvironment, TAMs exhibit a pro-tumor phenotype through the upregulation of glycolysis, fatty acid oxidation, cholesterol efflux, as well as arginine, tryptophan, glutamate, and glutamine metabolism (17). However, the relationship between cellular metabolism, especially norepinephrine metabolism and TAM activity in MPE remains unclear. Therefore, the aim of our study was to evaluate the crosstalk between cellular metabolism and macrophage immunosuppression and whether β-receptor blocker treatment prevents TAM immunosuppression by affecting norepinephrine metabolism in MPE.

## Methods

### Human samples

Samples were obtained from 132 patients with malignant and benign pleural effusions (MPE and BPE, respectively) at the First Affiliated Hospital of Zhengzhou University between April 2016 and May 2017. A total of 59 men and 73 women were included in this study. The average age was 58.02 years (range, 26-91 years). Among them, 82 and 50 patients were in the BPE and MPE groups, respectively. Of the 50 samples in MPE groups, 48 samples were from patients with lung cancer, 2 samples were from patients with malignant mesothelioma. The diagnostic criteria for BPE were as follows: 1. Tuberculous pleural effusion: adenosine deaminase > 45 U/L or a confirmed diagnosis of tuberculosis by thoracoscopy and histopathological biopsy, no evidence of MPE; 2. Pneumonic pleural effusion: Clinical observations suggesting the presence of pulmonary inflammation due to presumed infection (bacteria other than mycobacteria, viruses, fungi, or parasites) and corresponding clinical manifestations. The diagnostic criteria for MPE were clinical diagnosis of malignant tumor through histopathological examination, positive pleural effusion exfoliated cytology test, or significantly increased tumor markers in pleural effusion. A total of 6 patients had MPE and combined cardiovascular diseases (without prior β-blocker exposure) receiving β-receptor blockers as part of the standard of care for their heart disease. Patients received Computed Tomography (CT) examination as routine care to evaluate the condition and efficacy. The time frame of diagnosis and treatment in MPE and heart disease patients received propranolol was shown in **Supplementary Figure 1A**. The collection of specimens was approved by the institutional ethics committee of our hospital and informed consent was obtained from each patient with available follow-up information.

### Pleural effusion mouse model

Mice with malignant pleural effusion were purchased from Jiangsu Huachuang Sino Pharma Tech Co., Ltd. In brief, six- to eight-week-old male C57BL/6 mice were subcutaneously injected with 3 × 10^6^ Lewis lung carcinoma cells. When the tumor grew to a size of 50 mm^3^, the tumor was dissociated, digested into cell suspensions according to the method used for primary cell preparation, and then injected into the thoracic cavity of six- to eight-week-old male C57BL/6 mice at 200ul (18). After five days, twelve mice were randomized into two groups. Propranolol (40 mg/kg)/Saline was administered by intraperitoneal injection every day until the mice were sacrificed. Mice were sacrificed by cervical dislocation on day 16 after tumor cell implantation, and the pleural effusion was used for further analysis. All mice were kept in individually ventilated cages under specific-pathogen-free condition, and protocol was approved by the Experimental Animal Welfare Ethics Committee of the Experimental Animal Center of Zhengzhou University (NO. ZZU-LAC20240112[11]).

### Preparation of pleural effusion

MPE and BPE samples collected from the patients were centrifuged at 3000 rpm for 10 min. The supernatant was collected for quantitative determination of lactate and norepinephrine. After washing the compacted pellet three times with sterile phosphate-buffered saline (PBS), the pellets were digested with trypsin and filtered using a 70 µm cell strainer (BioLegend, San Diego, CA, USA). The cells were washed with RPMI 1640 medium supplemented with 10% fetal bovine serum (FBS) and resuspended in 10 mL of medium. Mononuclear cells in pleural effusion were collected by Ficoll-Paque density gradient centrifugation. The cell suspension was then added to the surface of the Ficoll-Paque smoothly to prevent sinking of the cell suspension and centrifuged at 2500 rpm for 25 min. Finally, the mononuclear cells were collected and isolated from the interlayer of the solution.

### Preparation of primary cell

The tumor tissue was washed three times with PBS, and the fat and connective tissue were removed. The tumor tissue was cut into small pieces (1-2 mm), washed three times with PBS, and digested with 0.25% trypsin at 37°C for 30 min, and gently shaken every 5 min. Digestion was terminated by adding serum containing medium, the suspension was filtered twice, centrifuged at 1000 rpm for 5-10 min, and the cells were resuspended in 200 uL of PBS.

### Monocyte isolation and cell induction

CD14^+^ cells were isolated from peripheral blood mononuclear cells using auto-MACS with human CD14 microbeads (Miltenyi Biotec) according to the manufacturer’s instructions. The CD14^+^ cells were resuspended at a final concentration of 1 **×** 10^6^/mL in RPMI 1640 medium supplemented with 10% FBS, 20 ng/mL M-CSF, 100 U/mL penicillin, and 100 g/mL streptomycin. On days 1, 3, and 5, the medium was changed by adding M-CSF (20 ng/mL) under normoxic conditions. L-(+)-Lactic acid (Sigma-Aldrich Cat# L6402; CAS: 79-33-4) (0, 12.5, and 25 mM) and tyrosine hydroxylase inhibitor (50 µM; H-Tyr (3-1)-OH; MCE​) were added on day 7 for 24 h under hypoxic conditions. Norepinephrine​ (10 µM; MCE​), isoproterenol (10 µM) and tyrosine hydroxylase inhibitor (50 µM) were added on day 7 for 24 h under normoxic conditions. The cells were cultured in normoxic (20% O_2_) and hypoxic (1% O_2_) incubators (Eppendorf, Galaxy48R).

### Flow cytometry analysis

Peripheral blood mononuclear cells were isolated, and cell surface molecules were stained for 20 min at 4 °C in the dark using saturating concentrations of the following antibodies: anti-human CD14, CD163, PD-L1, 7-AAD antibodies, and Zombie AquaTM Fixable Viability kit (BioLegend). The cells were then analyzed by flow cytometry (BD FACS Canto II, BD Biosciences; DxFLEX B5-R3-V5, Beckman Coulter). Mononuclear cells isolated from the MPE were first stained for 20 min at 4°C in the dark using saturating concentrations of the following antibodies: CD3, CD8, CD56, HLA/DR, CD11b, CD33, CD14, CD163, PD-L1 antibodies, and Zombie AquaTM Fixable Viability kit (BioLegend). For intracellular staining of arginase-1(ARG-1), phenylalanine hydroxylase (PAH), and tyrosine hydroxylase (TH), cells were fixed with 4% formalin for 30 min and incubated with permeabilization washing buffer for 30 min. Rabbit anti-ARG-1 (1:50; Cell Signaling Technology), rabbit anti-PAH, and rabbit anti-TH (1:100; Abcam) were used as the primary antibodies. Alexa Fluor 647-AffiniPure Donkey Anti-Rabbit IgG (1:300; Jackson ImmunoResearch) was used to detect the primary antibodies using flow cytometry (DxFLEX B5-R3-V5, Beckman Coulter). In addition, the percentages of TAMs and TAM-affected killing of tumor cells by NK cells before and after treatment with lactate were analyzed using flow cytometry. For T cell function, cells were incubated with antibodies against intracellular targets including Perforin, IFN-γ, TNF-α antibodies, followed by surface staining with anti-human CD8 antibody. Mouse pleural effusion cells were first stained with surface target antibodies (CD45, CD3, CD8, CD4, NK1.1, CD11b, F4/80, GR1) and Zombie dye in the dark. Then the intracellular targets (perforin, IFN-γ, PD-L1, ARG-1, TNF-α) were incubated as above.

### Immunofluorescence staining

Cells were fixed with 4% paraformaldehyde and then permeabilized in 1% Triton X‐100 in PBS. Thereafter, the cells were blocked with bovine serum albumin (BSA) for 30 min and incubated with the following primary antibodies: rabbit anti-ARG-1 (1:50; Cell Signaling Technology), rabbit anti-PD-L1 (1:500; Abcam), rabbit anti-PAH and rabbit anti-TH (1:100; Abcam), mouse anti-PD-L1 (1:500; Proteintech, China), and mouse anti-ARG-1 (1:200; Biorbyt, UK) overnight at 4°C. Subsequently, the cells were incubated with the indicated secondary antibodies (1:300; Jackson ImmunoResearch) for 1 h at room temperature (RT) in the dark. Nuclei were stained with 4’,6‐diamidino‐2‐phenylindole (DAPI) for 5 min at RT. The cells were imaged at 4× and 20× magnification using a Vectra automated multispectral microscope, and the InForm Image Analysis software was used to generate a spectral library for unmixing (both from PerkinElmer).

### NK cell-mediated cytotoxicity assay

A549 cells (1×10^5^ cells/mL) were incubated with carboxyfluorescein diacetate succinimidyl ester (CFSE) at 5nM concentration for 30 min in the dark and washed 3 times with complete medium. Macrophages treated with or without propranolol, NK-92 cells and A549 cells were co-incubated at the indicated ratios of 1:1, 5:1, 10:1 and 20:1 in 96-well plates at 37°C. 6 h later, the cells were trypsinized, washed with PBS for 3 times, and re-suspended in binding buffer, followed by staining using propidium iodide (PI) kits. Then the cells were washed twice with PBS and detected using a FACS Calibur flow cytometer (BD Biosciences, Franklin Lakes, NJ, USA). The data were analyzed using FlowJo software. The efficacy of lysis was obtained as the total number of CFSE and PI double positive killed target cells divided by the total number of target cells.

### T cell suppression assay

CD8^+^ T cells were isolated from peripheral blood mononuclear cells using auto-MACS with human CD8 microbeads. As for the effect of propranolol on T cell activation impaired by TAMs, T cells were incubated with propranolol-treated TAMs *in vitro* at a 4:1 ratio for 24 h. T cell function for detecting the expressions of perforin, IFN-γ and TNF-α was analyzed using flow cytometry.

### Quantitative determination of lactate and norepinephrine

Lactate concentrations in the BPE and MPE samples were measured using a Lactate Colorimetric/Fluorometric Assay kit (Biovision) and Lactate Assay Kit (Cell Biolabs) and according to the manufacturer’s instructions and read immediately at 570 nm (Molecular Devices, SpectraMax iD3).

Norepinephrine concentrations in BPE and MPE were measured using a noradrenaline/norepinephrine (NA/NE) ELISA kit (Abbexa) according to the manufacturer’s instructions and read at 450 nm (Molecular Devices, SpectraMax iD3).

### Cell transfection

CD14^+^ monocytes were induced to differentiate into M2-like macrophages on day 7, and then transfected with negative control siRNA or siRNA targeting PAH (siPAH) purchased from Shanghai GenePharma Co., Ltd. using jetPRIME reagent (Polyplus-transfection SA). After 4 h, the medium was changed, and cells were further incubated with different concentrations of lactate (0, 12.5, and 25 mM) in a hypoxic incubator for 24 h to simulate the hypoxia microenvironment of malignant pleural effusion. The sequences for the sense and antisense strands of the PAH siRNA and negative control siRNA are provided below.

#### siPAH1

sense (5’ to 3’): GCGCUUAUUUGAGGAGAAUTT

antisense (5’ to 3’): AUUCUCCUCAAAUAAGCGCTT

#### siPAH2

sense (5’ to 3’): GUGGCUUCCAUGAAGAUAATT

antisense (5’ to 3’): UUAUCUUCAUGGAAGCCACTT

#### Negative control

sense (5’ to 3’): UUCUCCGAACGUGUCACGUTT

antisense (5’ to 3’): ACGUGACACGUUCGGAGAATT

### Metabolomic analysis

UPLC-MS was used to analyze the differences in metabolites between MPE, BPE, and macrophages under different lactate concentrations (0, 12.5, and 25 mM) under hypoxia (1% O_2_) or normoxia (20% O_2_). Thermo Scientific Q Exactive (Quadrupole-orbitrap mass Spectrometer) and Dionex UltiMate 3000 were used in this study. The chromatographic columns were waters Acquity UPLC HSS T3 column (2.1 × 100 mm, 1.8 μm) and Acquity UPLC BEH C18 column (2.1 × 50 mm, 1.7 μm). The data were imported into the SIEVE2.2 analysis for peak area normalization. Using the software SIMCA 14.0 for multivariate statistical analysis, PCA and OPLS-DA models were used to analyzve the data. SPSS 19.0 software was used for t tests to screen out potential differential metabolites (VIP > 1, P < 0.05). We used small molecule screening software (OSI/SMMSTM) and online databases (Metlin, HMDB, MZ-Cloud, etc.) to determine the precise molecular weight and possible ion-binding forms. The structures of the different metabolites were confirmed using MS and MS2. We used Kyoto Encyclopedia of Genes and Genomes (KEGG) to analyze the pathways in which the differential compounds were involved.

### Quantitative real-time PCR

Total RNA was extracted using TRIzol reagent (Invitrogen, USA). The RNA quality and concentration were determined using a NanoDrop 2000 spectrophotometer (Thermo Fisher Scientific). For amplification, 1 µg of total RNA was reverse transcribed to cDNA, followed by incubation at 37°C for 15 min and 85°C for 5 s, according to the manufacturer’s protocol (Takara Biotechnology). qPCR was performed using a real-time PCR system (Agilent Stratagene). The PCR conditions were as follows: 95°C for 10 min; 40 cycles of 95°C for 10 s, 60°C for 10 s, and 72°C for 10 s. The relative expression of target genes was determined by normalizing the expression of each target gene to that of β-actin. The data were analyzed using the 2^-ΔΔCt^ method. (**Supplementary Table S1** provides primer sequences used).

### RNA-seq

Total RNA from macrophages treated with different lactate concentrations (0, 12.5, and 25 mM) and propranolol under hypoxic conditions (1% O_2_) was isolated using TRIzol reagent (Invitrogen) according to the manufacturer’s instructions. RNA-seq was performed by the Wuhan BGI Technology Service company using the MGISEQ-200 platform. Transcript analysis was performed using Dr.Tom 2.0 (https://biosys.bgi.com).

### Western blotting

Total protein was extracted by lysing the cells in RIPA lysis buffer supplemented with protease inhibitor cocktail (P8340; Sigma-Aldrich) and phosphatase inhibitor cocktail 2 (P5726; Sigma-Aldrich). The following primary antibodies were used: rabbit anti-phospho-ERK, rabbit anti-ARG-1 (1:1000; Cell Signaling Technology), rabbit anti-PD-L1 (1:1000; Abcam), and mouse anti-β-actin (1:2000; Cell Signaling Technology). Primary antibodies were detected using HRP-conjugated goat polyclonal rabbit or mouse IgG antibodies (1:5000; Cell Signaling Technology) and enhanced chemiluminescence.

### Statistics

The results were analyzed using GraphPad Prism 9.5.1 (GraphPad Software, La Jolla, CA, USA). In bar and dot graphs, bars indicate means and error bars indicate SD. Significance was assessed by Student’s two-tailed test to determine the significance of the difference between means of two groups. One-way ANOVA was used to compare means among three or more in dependent groups. Tukey’s *post hoc* tests were used to compare all pairs of treatment groups when the overall p value was <0.05. A stepwise conditional logistic regression model was used for multivariate regression analysis to analyze the relationship between lactate levels and M2-like macrophages in patients with MPE or BPE. A total of 227 lung adenocarcinoma samples from The Cancer Genome Atlas (TCGA) dataset (http://cancergenome.nih.gov/) were included. All lung adenocarcinoma tissue samples were divided according to the expression of high or low levels of LDH. The differentially expressed genes were screened using the R language and the edgeR package with a difference of more than two times, and then the differential pathways, based on the differentially expressed genes, were analyzed using GO (http://www.geneontology.org/) and KEGG. The level of significance was set at P < 0.05.

## Results

### MPE patients treated with β-receptor blocker show a reduction in MPE volumes by reversing macrophage immunosuppression

**Figure 1:**
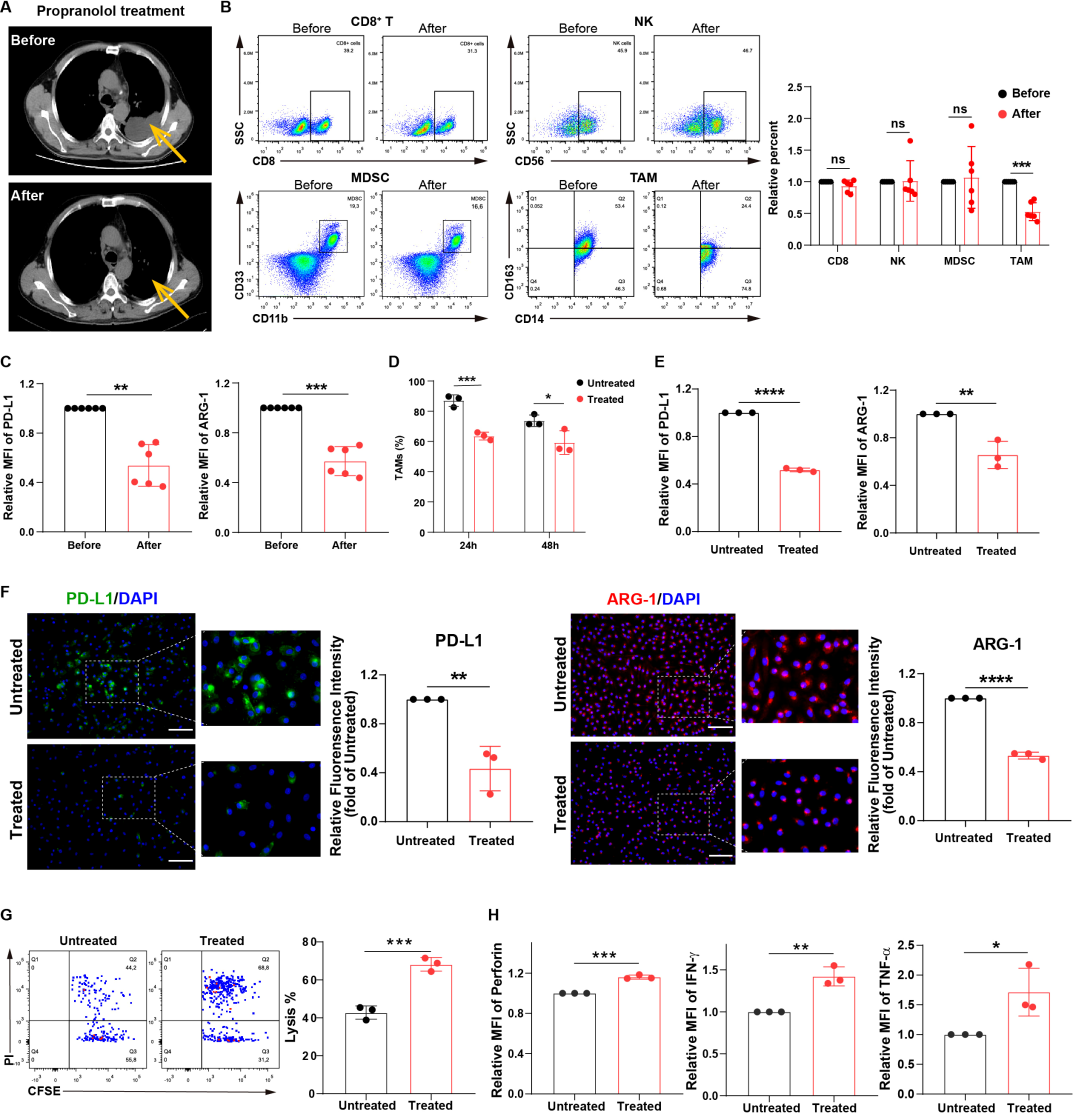
MPE patients treated with β-receptor blocker show a reduction in MPE volumes owing to the inhibition of macrophage immunosuppression. **(A)** CT imaging showing the effusion volume change in MPE patients with heart disease (n=6) before and after treatment with propranolol (30 mg/day). One respective CT imaging was shown. Yellow arrows indicate effusion volumes. The percentages of immune cells, including CD8+ T, NK, MDSC, and TAM cells **(B)**, and the expression of PD-L1 and ARG-1 in TAMs **(C)** before and after treatment with propranolol (5-10 mg/dose/time, three times/day, for three consecutive days orally) in MPE patients with heart disease (n=6) were analyzed using flow cytometry. **(D)** The percentages of CD163+ TAMs before and after treatment with propranolol (10 µM) *in vitro* were analyzed using flow cytometry. The expression of PD-L1 and ARG-1 in macrophages before and after treatment with propranolol (10 µM) *in vitro* was analyzed using flow cytometry **(E)** and immunofluorescence **(F)**. **(G)** TAM-affected NK cell-mediated apoptosis of tumor cells with and without propranolol treatment (10 µM) *in vitro* was analyzed using flow cytometry. **(H)** TAM-affected T cell dysfunction with and without propranolol treatment (10 µM) *in vitro* was analyzed using flow cytometry. MFI, median fluorescence intensity. *P < 0.05, **P < 0.01, ***P < 0.001, ****P < 0.0001 and ns = no significance.

We found that in patients with MPE and heart disease, treatment with β-receptor blocker, propranolol, demonstrated a significant decrease in effusion volumes (**Figure 1A**) and an improved condition. Furthermore, we observed that among CD8^+^ T cells, NK cells, myeloid-derived suppressor cells (MDSCs), and TAMs, the TAM frequency and the levels of immunosuppressive markers PD-L1 and arginase-1 (ARG-1) in TAMs were decreased after treatment with propranolol in these MPE samples (**Figures 1B, C**; **Supplementary Figures 1B–F**). Next, MPE samples were acquired and treated with β-receptor blocker *ex vivo* to further evaluate the changes in immune cell frequency. We observed that the TAM frequency was decreased after propranolol treatment, while other immune cell frequency change was no significant (**Supplementary Figure 1G**). In addition, our previous study indicated TAMs were the dominant immunosuppressive cell population in MPE (19). Based on the results from our current and previous studies, we further analyzed the changes in induced M2-like macrophages (TAMs) before and after treatment with β-receptor blocker *in vitro*. TAM frequency was significantly decreased after propranolol treatment *in vitro* (**Figure 1D**). Moreover, we investigated the immunosuppressive phenotype of TAMs before and after treatment with β-receptor blocker. Propranolol significantly decreased the expression of immunosuppressive proteins PD-L1 and ARG-1 in macrophages (**Figures 1E, F**). Furthermore, treatment with propranolol reversed TAM-induced impairment of the anti-tumor effects of NK cells (**Figure 1G**; **Supplementary Figure 1H**) and T cells (**Figure 1H**). These data suggest treatment with β-receptor blocker may improve MPE outcome by downregulating TAM frequency and immunosuppression, thereby enhancing anti-tumor immunity.

### Macrophages exhibit an immunosuppressive phenotype via the activation of norepinephrine metabolism

**Figure 2:**
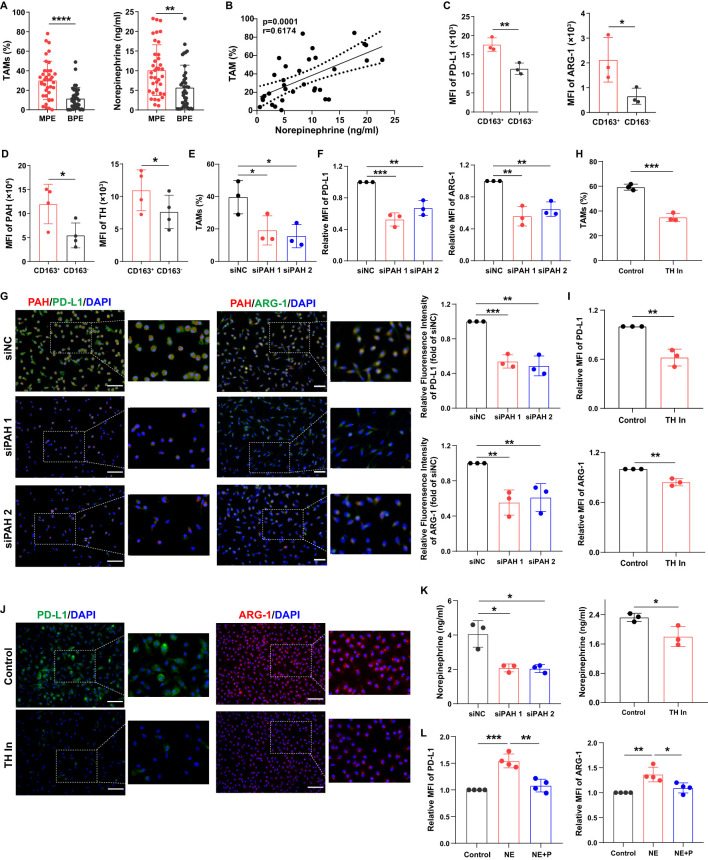
Macrophages exhibit an immunosuppressive phenotype via the activation of norepinephrine metabolism. **(A)** The percentages of TAMs in MPE (n=38) and BPE (n=38) were analyzed using flow cytometry, and the levels of norepinephrine in MPE and BPE were analyzed using ELISA. **(B)** The correlation between TAM frequency and norepinephrine levels in MPE was analyzed. The expression of PD-L1 and ARG-1 **(C)**, PAH and TH **(D)** in CD163^+^ and CD163^-^ macrophages sorted from MPE was analyzed using flow cytometry. After the knockdown of PAH using siRNA, the percentage of TAMs was analyzed using flow cytometry **(E)**, and the expression of PD-L1 and ARG-1 in macrophages was analyzed using flow cytometry **(F)** and immunofluorescence **(G)**. After treatment with the TH inhibitor (50 µM), the percentage of TAMs was analyzed using flow cytometry **(H)**, and the expression of PD-L1 and ARG-1 in macrophages was analyzed using flow cytometry **(I)** and immunofluorescence **(J)**. **(K)** The secretion of norepinephrine by macrophages was analyzed after blockade of PAH and TH. **(L)** The expression of PD-L1 and ARG-1 in macrophages treated with norepinephrine and propranolol *in vitro* was analyzed using flow cytometry. MFI, median fluorescence intensity. *P < 0.05, **P < 0.01, ***P < 0.001, ****P < 0.0001.

Propranolol is a β-receptor blocker (11) that inhibits norepinephrine signaling. Norepinephrine is a product of phenylalanine, tyrosine, and dopamine metabolism. Therefore, we hypothesized that β-receptor blocker, propranolol can influence macrophage function by inhibiting norepinephrine-related signaling pathways. We analyzed the percentage of TAMs and norepinephrine levels in MPE and benign pleural effusion (BPE). The results showed that TAM frequency was markedly higher in MPE than that in BPE, and the levels of norepinephrine in MPE were significantly increased (**Figure 2A**). Furthermore, TAM frequency was closely associated with norepinephrine levels in the MPE group (**Figure 2B**).

To evaluate whether phenylalanine, tyrosine, and dopamine signaling mediated macrophage immunosuppression, we analyzed the expression of their related metabolic enzymes. We found that MPE-derived CD163^+^ macrophages exhibited an immunosuppressive phenotype with higher levels of PD-L1 and ARG-1 compared to CD163^-^ macrophages (**Figure 2C**). Phenylalanine hydroxylase (PAH), a crucial enzyme that converts phenylalanine into tyrosine, and tyrosine hydroxylase (TH), a critical enzyme that catalyzes the conversion of tyrosine to dopamine (20, 21), were increased in MPE-derived CD163^+^ macrophages (**Figure 2D**). After PAH knockdown, the percentage of TAMs (**Figure 2E**) and the expression of PD-L1 and ARG-1 in TAMs (**Figures 2F, G**) were significantly decreased *in vitro*. After blocking TH, TAM frequency (**Figure 2H**) and PD-L1 and ARG-1 expression in TAMs (**Figures 2I, J**) were also decreased *in vitro*. In addition, after blockade of PAH and TH, norepinephrine secretion in macrophages was decreased (**Figure 2K**). The immunosuppressive phenotype molecules in macrophages were upregulated after norepinephrine treatment, but was impaired after propranolol treatment (**Figure 2L**). These findings indicate that macrophages exhibit an immunosuppressive phenotype via the activation of norepinephrine metabolism.

### Lactate is closely associated with alternatively activated macrophages in MPE

**Figure 3:**
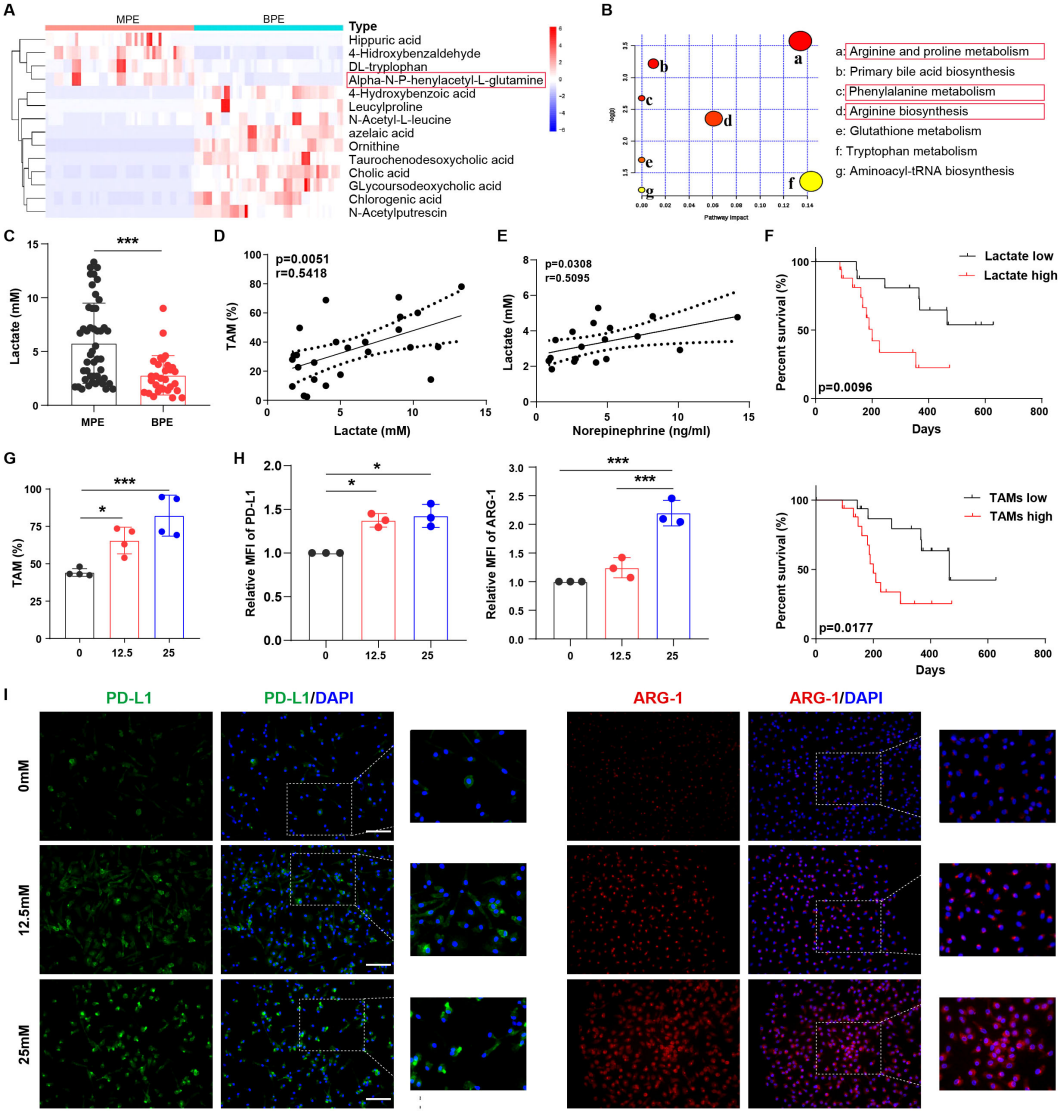
Lactate is closely associated with alternatively activated macrophages in MPE. **(A)** Heat map showing the top dominant differential metabolites in MPE and BPE as determined by mass spectrometry. **(B)** Bubble chart showing the enriched top differential metabolic signaling pathways in MPE. **(C)** Lactate levels in MPE (n=47) and BPE (n=30) were analyzed using ELISA. **(D)** The correlation between TAM frequency and lactate levels in MPE was analyzed. **(E)** The correlation between norepinephrine and lactate levels in MPE was analyzed. **(F)** Survival of patients with high and low levels of lactate or TAMs was analyzed. **(G)** The percentage of lactate-treated TAMs (0, 12.5, and 25 mM) under hypoxia was analyzed using flow cytometry. The expression of PD-L1 and ARG-1 in lactate-treated macrophages (0, 12.5, and 25 mM) under hypoxic conditions was analyzed by flow cytometry **(H)** and immunofluorescence **(I)**. MFI, median fluorescence intensity. *P < 0.05, ***P < 0.001.

To further investigate the factors that may influence macrophage immunosuppression in MPE, we assessed the differential metabolites between MPE and BPE (**Supplementary Figures 2A-D**). Glutamine levels were significantly increased in the MPE group (**Figure 3A**). Furthermore, the metabolism of phenylalanine and arginine was upregulated in MPE (**Figure 3B**; **Supplementary Figure 2E**). This suggests that phenylalanine and arginine metabolism might contribute to the immunosuppressive function of TAMs. The tumor microenvironment is under hypoxic condition with abundant lactate (22, 23). Therefore, we conducted an analysis of lactate levels in the effusion and observed a significant higher concentration in MPE (**Figure 3C**). Lactate is classically an end product of glycolysis, but can also be one of the downstream products of glutamine (24). The elevated lactate was consistent with the elevated glutamine, which suggested a potential source of lactate from glutamine metabolism. Moreover, there was a close positive correlation between TAM frequency and lactate levels in MPE (**Figure 3D**), whereas no significant correlation between CD163^+^ macrophages and lactate levels was observed in BPE (**Supplementary Figure 2F**). In addition, lactate levels were closely associated with norepinephrine levels in MPE (**Figure 3E**), and patients with high levels of lactate or TAMs in MPE had poor survival rates (**Figure 3F**). These data demonstrate that lactate may contribute to the increase in TAM frequency and inhibition of anti-tumor immunity in MPE.

In order to simulate the nature environment of MPE, we investigated the effect of lactate on macrophages under hypoxia. *In vitro* experiments showed that lactate increased TAM frequency (**Figure 3G**) and CD163 expression in macrophages (**Supplementary Figure 2G**) under hypoxia, whereas no significant effect on TAM frequency was observed under normoxia (**Supplementary Figure 2H**). Furthermore, under hypoxic conditions, lactate induced an immunosuppressive phenotype in macrophages with enhanced levels of PD-L1, ARG-1 (**Figures 3H, I**; **Supplementary Figures 2I, J**), IL-6, and VEGF (**Supplementary Figures 2K, L**). However, under normoxia, lactate did not elevate immunosuppressive biomarker IL-6 and VEGF in macrophages, except for PD-L1 and ARG-1, which were only slightly elevated compared to those in macrophages under hypoxia (**Supplementary Figures 2I-L**). Collectively, lactate is closely associated with the percentage and function of alternatively activated macrophages in MPE.

### Lactate triggers phenylalanine/norepinephrine signaling to induce macrophage immunosuppression

**Figure 4:**
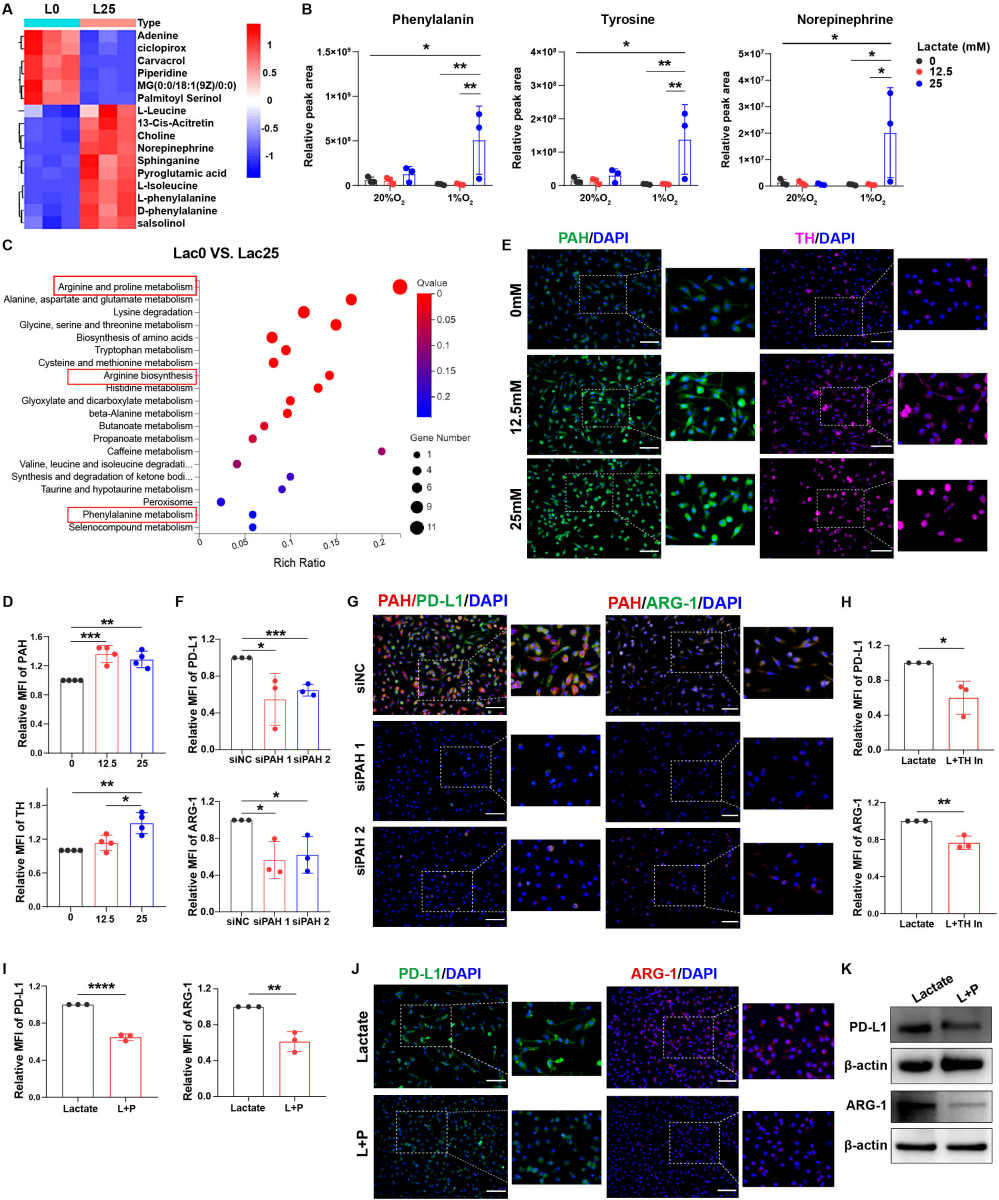
Lactate triggers phenylalanine/tyrosine/norepinephrine signaling to induce macrophage immunosuppression. **(A)** Heat map showing the cellular metabolism in macrophages before and after treatment with lactate (25 mM). **(B)** Relative peak areas of phenylalanine, tyrosine, and norepinephrine were analyzed using mass spectrometry. **(C)** Bubble chart showing RNA-seq analysis of macrophages before and after treatment with lactate (25 mM) under hypoxia. The expression of PAH and TH in lactate-treated macrophages (0, 12.5, and 25 mM) under hypoxic conditions was analyzed using flow cytometry **(D)** and immunofluorescence **(E)**. The expression of PD-L1 and ARG-1 in lactate-treated macrophages before and after transfection with siRNA-PAH under hypoxic conditions was analyzed using flow cytometry **(F)** and immunofluorescence **(G)**. **(H)** The expression of PD-L1 and ARG-1 in lactate-treated macrophages under hypoxia before and after treatment with a TH inhibitor (50 µM) was analyzed using flow cytometry. The expression of PD-L1 and ARG-1 in lactate-treated macrophages under hypoxia before and after treatment with propranolol (10 µM) was analyzed using flow cytometry **(I)**, immunofluorescence **(J)**, and western blotting **(K)**. MFI, median fluorescence intensity. *P < 0.05, **P < 0.01, ***P < 0.001, ****P < 0.0001.

To further investigate whether macrophage immunosuppression mediated by phenylalanine, tyrosine, and norepinephrine signaling was triggered by lactate, we analyzed the differences in metabolites in macrophages before and after lactate treatment under normoxia or hypoxia using mass spectrometry (**Supplementary Figure 3A**). Phenylalanine and norepinephrine metabolism were activated in lactate-treated macrophages under hypoxic conditions (**Figure 4A**; **Supplementary Figures 3B-D**). Furthermore, levels of phenylalanine, tyrosine, and norepinephrine were significantly higher in lactate-treated macrophages under hypoxic conditions (**Figure 4B**; **Supplementary Figures 3E, F**). The metabolic signaling pathways of phenylalanine and arginine were also activated in lactate-treated macrophages under hypoxic conditions using RNA sequencing (RNA-seq; **Figure 4C**; **Supplementary Figures 3G, H**). We then analyzed the expression of enzymes related to this metabolic pathway and found that PAH, TH, dopamine decarboxylase (DDC), and dopamine beta hydroxylase (DBH) levels were remarkably increased in lactate-treated macrophages under hypoxic conditions (**Figures 4D, E**; **Supplementary Figures 3I, J**).

After the knockdown of PAH expression in macrophages using small interfering RNA (siRNA) (**Supplementary Figures 4A, B**), PD-L1 and ARG-1 levels were decreased in lactate-treated macrophages under hypoxic conditions (**Figures 4F, G**; **Supplementary Figures 4C, D**). After TH blockade, the levels of PD-L1 and ARG-1 were also significantly decreased (**Figure 4H**; **Supplementary Figures 4E-G**). Treatment with β-receptor blocker to inhibit norepinephrine signaling resulted in decreased PD-L1, ARG-1, and CD163 expression in lactate-treated macrophages (**Figures 4I-K**; **Supplementary Figures 4H-J**). Therefore, lactate induces macrophage immunosuppression via activation of phenylalanine, tyrosine, and norepinephrine metabolism.

### Lactate mediated-immunosuppression is depended on ERK in macrophages

**Figure 5:**
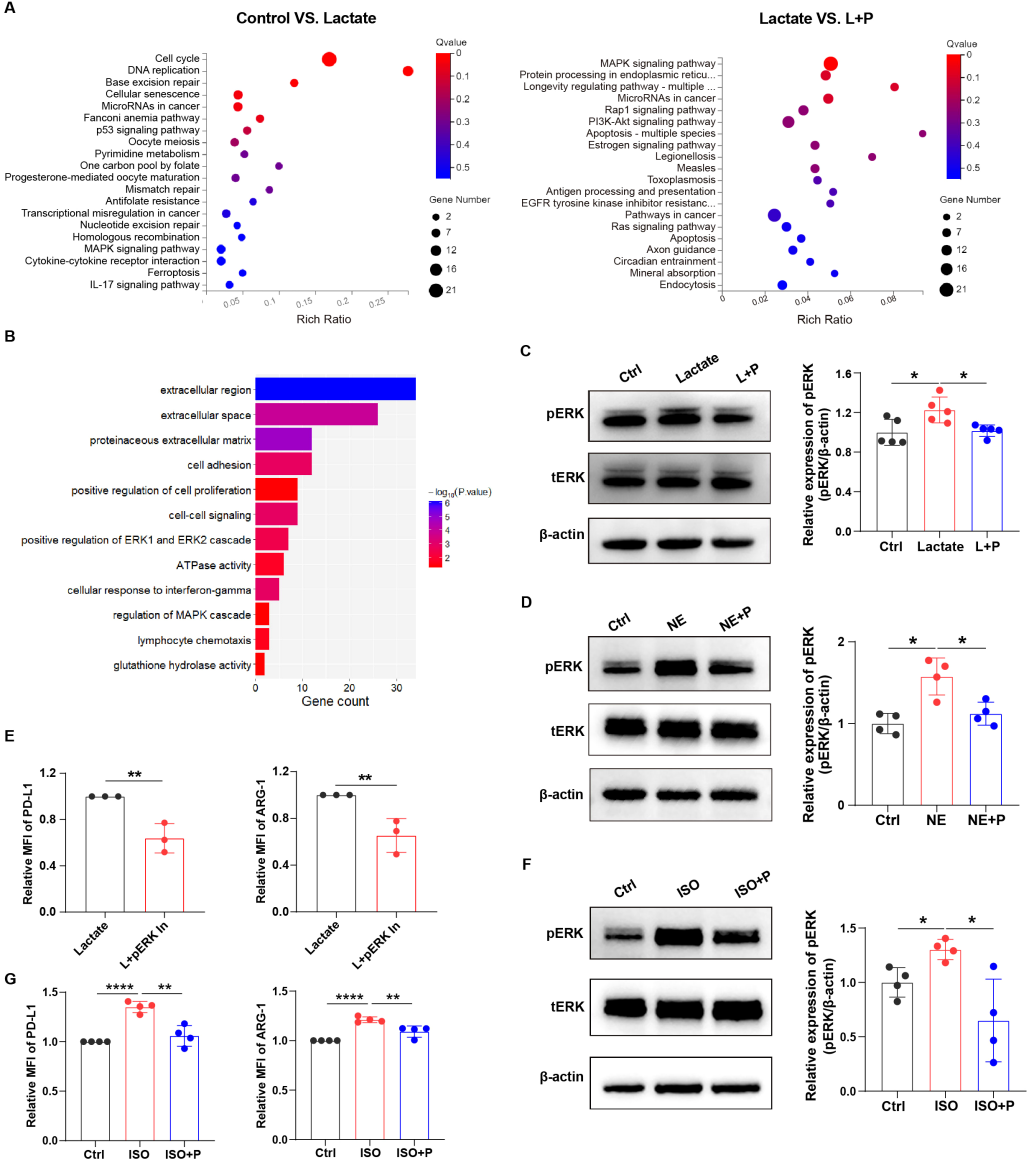
Lactate mediated-immunosuppression is depended on ERK in macrophages. **(A)** Bubble chart showing the RNA-seq results in macrophages with or without lactate treatment (12.5 mM) under hypoxia (left); and the RNA-seq results in lactate-treated macrophages with or without propranolol treatment (right). **(B)** Bar chart showing the enriched signaling pathways in lung cancer patients with high levels of LDH. **(C)** The protein expression of pERK in lactate-treated macrophages before and after treatment with propranolol (10 µM) was analyzed using western blotting. The protein expression of pERK in macrophages treated with norepinephrine (NE, 10 µM) **(D)** and isoproterenol (ISO, 10 µM) **(F)** with or without propranolol treatment (10 µM) was analyzed using western blotting. **(E)** The expression of PD-L1 and ARG-1 in lactate-treated macrophages before and after treatment with a pERK inhibitor (4 nM; SCH772984, Selleckchem) was analyzed using flow cytometry. **(G)** The expression of PD-L1 and ARG-1 in isoproterenol-treated macrophages with or without propranolol treatment (10 µM) using flow cytometry. MFI, median fluorescence intensity. *P < 0.05, **P < 0.01, ****P < 0.0001.

The MAPK signaling pathway, which can be divided into three types (ERK, JNK, and p38MAPK) (25, 26), was activated in lactate-treated macrophages (**Figure 5A**). Lactate dehydrogenase (LDH) is a key enzyme that indirectly affects lactate levels (23). Therefore, we performed pathway enrichment analysis in lung cancer patients with high LDH expression using sequencing results from the TCGA database, and found that ERK signaling was enriched (**Figure 5B**; **Supplementary Figure 4K**). We further investigated whether norepinephrine-mediated lactate-induced macrophage immunosuppression depends on ERK. After blocking norepinephrine signaling using propranolol, lactate-mediated activation of ERK signaling in macrophages was inhibited (**Figure 5C**). And norepinephrine also triggered ERK signaling, which was inhibited after propranolol treatment (**Figure 5D**). Furthermore, ERK inhibitor treatment decreased the levels of PD-L1 and ARG-1 in lactate-treated macrophages (**Figure 5E**), suggesting that lactate-treated macrophage immunosuppression depends on ERK signaling. It appears that norepinephrine mediates macrophage immunosuppression through activation of ERK signaling pathway by β-receptor. Next, we used the β-receptor agonist isoproterenol to further demonstrate that the β-receptor signaling pathway does indeed promote macrophage immunosuppression by activating the ERK signaling pathway (**Figures 5F, G**). Collectively, these data suggest that lactate triggers phenylalanine/norepinephrine signaling to induce macrophage immunosuppression through ERK.

### β-receptor blocker treatment inhibits MPE development by enhancing anti-tumor immune response *in vivo*

**Figure 6:**
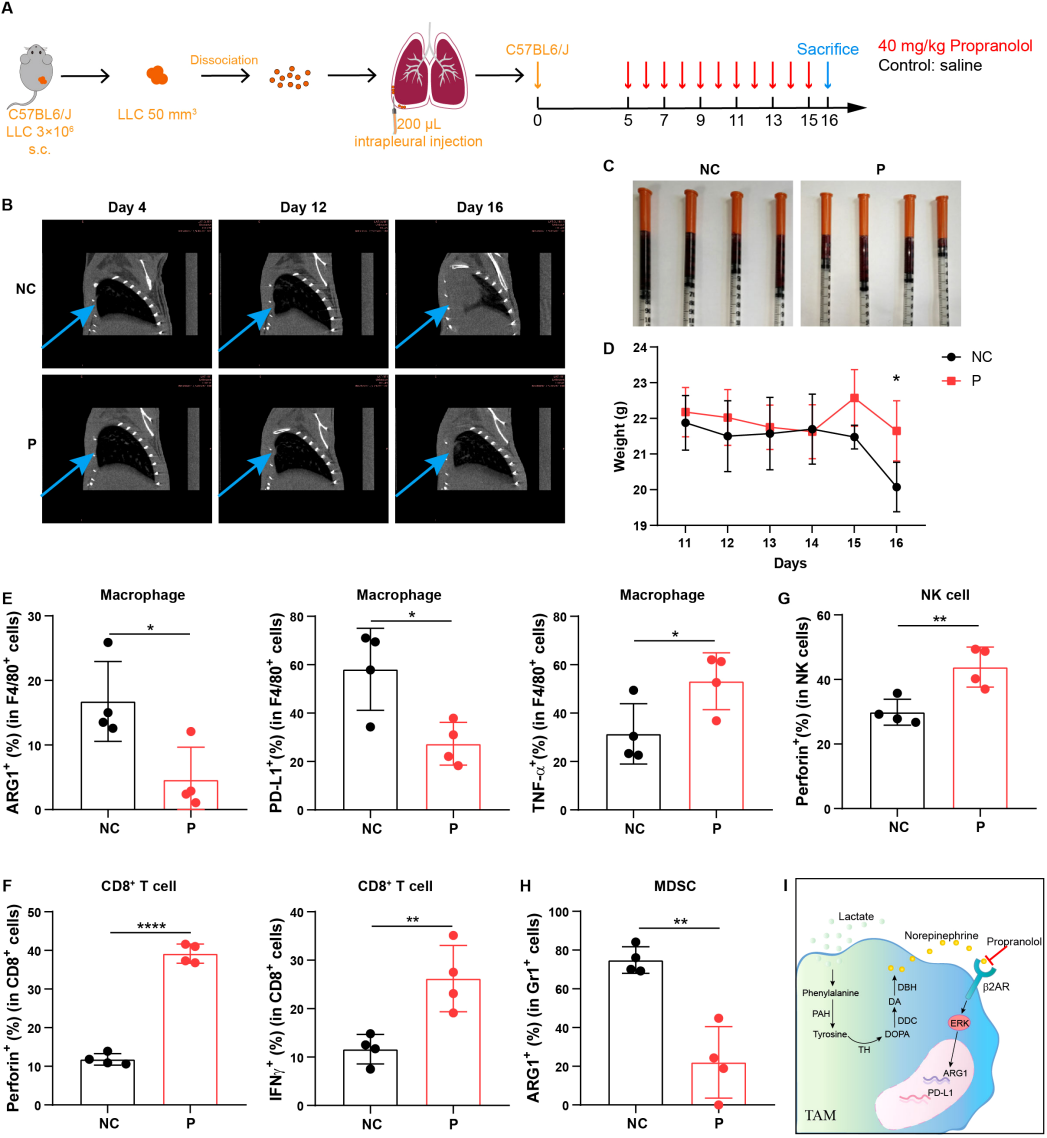
β-receptor blocker treatment inhibits MPE development by enhancing anti-tumor immune response *in vivo*. **(A)** Schematic of MPE model design and β-receptor blocker, propranolol treatment in mouse model. **(B)** Chest CT imaging of MPE mice with or without propranolol treatment. The blue arrows indicate tumors located in the pleural cavity in mouse model. **(C)** MPE volume in mice administered with or without propranolol treatment (n = 4 mice). **(D)** Body weight of MPE mice administered with or without propranolol treatment (n = 4 mice). Percentage of ARG1^+^, PD-L1^+^, and TNF-α^+^ cells in F4/80^+^ macrophages **(E)**, perforin^+^ and IFN-γ^+^ cells in CD8^+^ T cells **(F)**, perforin^+^ cells in NK cells **(G)**, and ARG1^+^ cells in Gr1^+^ cells **(H)** in MPE with or without propranolol treatment. **(I)** Mechanism diagram through which lactate mediates macrophage immunosuppression via the activation of phenylalanine, tyrosine and norepinephrine signaling. *P < 0.05, **P < 0.01, ****P < 0.0001.

To further investigate the efficacy of β-receptor blocker in the treatment of MPE, we developed a mouse model of MPE by injecting Lewis lung carcinoma (LLC) cells directly into pleural cavity of C57BL/6 mice. Then we administrated propranolol into MPE-bearing mice once a day from day 5 to day 15 after LLC injection (**Figure 6A**). We found that with the development of MPE, the pleural tumor burden and pleural effusion was gradually formed (**Figures 6B, C**; **Supplementary Figure 5A**); however, after the treatment with propranolol, the tumor burden was reduced (**Figure 6B**) and pleural effusion volume was decreased (**Figure 6C**; **Supplementary Figure 5B**). Moreover, the body weight of mouse was significantly higher in propranolol-treated group compared to control group (**Figure 6D**). Furthermore, we evaluated the changes in the immune microenvironment before and after the treatment with β-receptor blocker. Although the immune cell frequency in MPE before and after propranolol treatment was not significantly changed (**Supplementary Figures 5C-G**), we found that β-receptor blocker decreased the percentages of ARG1^+^ and PD-L1^+^ macrophages and increased the percentage of TNF-α^+^ macrophages in the effusion (**Figure 6E**; **Supplementary Figure 5J**). The effective function of T and NK cells was enhanced (**Figures 6F, G**; **Supplementary Figures 5H, I**) and the immunosuppression of MDSCs was obviously decreased (**Figure 6H**; **Supplementary Figure 5J**) after β-receptor blocker treatment. Together, these findings suggest that β-receptor blocker treatment inhibits tumor development by reversing the immunosuppressive MPE to enhance anti-tumor immune response.

## Discussion

Propranolol is a β-receptor blocker reported to have membrane-stabilizing properties but does not possess intrinsic sympathomimetic activity (11). Propranolol hydrochloride is used to treat hypertension, pheochromocytoma, myocardial infarction, cardiac arrhythmia, angina pectoris, and hypertrophic cardiomyopathy. It is also used to control the symptoms of sympathetic overactivity in hyperthyroidism, anxiety disorders, and tremors (27). Propranolol is also used as prophylaxis for migraine and upper gastrointestinal bleeding in patients with portal hypertension. In this study, we found and examined the effects of propranolol treatment in patients received propranolol for these aforementioned heart diseases and had MPE, suggesting that propranolol could be used as a novel therapeutic strategy for MPE patients.

Metabolic changes have been shown to affect myeloid cell immunosuppression and anti-tumor immunity (28–32). Aerobic glycolysis, a hallmark of cancer, can regulate MDSC immunosuppression and tumor immunity via a specific CEBPB isoform in triple-negative breast cancer (33). However, there are no reports on the relationship between amino acids, norepinephrine metabolism, and macrophage function in the tumor microenvironment. In this study, we demonstrated that lactate-induced phenylalanine, tyrosine, and norepinephrine signaling regulates macrophage immunosuppression in MPE. We performed untargeted metabolomics analysis on pleural effusion, which can conduct a comprehensive and systematic analysis of metabolites, but the results showed that the content of glycolytic metabolites was relatively low, probably because the mass spectrometry machine of untargeted metabolomics detection had a low resolution and could not well quantify the substances with low content and small molecular weight.

Lactic acid is a metabolic product commonly found in various physiological and pathological conditions, particularly in situations of anaerobic metabolism. It is known to be acidic, which can alter the local microenvironment. The acidity of this environment can influence cellular behavior, including the activation and differentiation of immune cells like macrophages. Tumor acidity contributes to prostate carcinogenesis by altering the state of macrophage activation (34). The acidity of the tumor microenvironment is a well-documented feature of many solid tumors and is known to promote TAM recruitment and polarization towards a tumor-promoting phenotype. Lactic acid itself can act as a signaling molecule, influencing metabolic pathways and gene expression in immune cells. Although acidic conditions can affect the tumor microenvironment, the effect of lactic acid itself on macrophage confirmed in this paper does exist, and there is support in the review (35). In this review, the authors argued that lactic acid itself can also promote the polarization of macrophages towards the M2 phenotype. Our findings revealed that the concentration of lactate was higher in malignant pleural effusion compared to benign pleural effusion. Therefore, we incorporated lactate into *in vitro* experiments to emulate the microenvironment of malignant pleural effusion. Although it is documented that the introduction of lactic acid can alter the pH value, our study did not specifically investigate whether the immunosuppressive signature observed in TAMs was attributed to the acidity or lactate itself.

Owing to increased glycolysis, lactate secretion is high in tumors, and this has immunosuppressive effects on the tumor microenvironment (24, 36, 37). Lactate is a critical immunoregulatory molecule involved in the inhibition of immune effector cell proliferation and induction of immune cell de-differentiation, resulting in anti-tumor immune escape and activation of innate and adaptive immune suppressor cells (37, 38). In addition, lactate mediates the suppression of effector T cell function and proliferation, but does not significantly alter regulatory T cells (37–41). Lactate modulates CD4^+^ T-cell polarization and induces an immunosuppressive tumor environment, which sustains the progression of prostate carcinoma via the Toll-like receptor 8 signaling pathway (42). In the current study, we also found that lactate promoted the immunosuppression of macrophages via the activation of phenylalanine, tyrosine, and norepinephrine metabolism, thereby promoting an immunosuppressive microenvironment in MPE. We detected that the concentration of lactate in the pleural effusion ranged from 0 to 15mM. To better understand the impact of high lactate concentration on the tumor immune microenvironment, we used different concentrations of lactate (0, 12.5, and 25 mM) to treat the cells (43). We noted that the presence of lactic acid at concentrations of both 12.5mM and 25mM significantly enhanced the immunosuppressive activity of tumor-associated macrophages. Moreover, our data showed that lactate plus hypoxic conditions markedly upregulated PD-L1 and Arg-1, suggesting that both lactate and hypoxia are required to promote the immunosuppression of macrophages. However, lactate treatment under normoxic condition had only a relatively weak increase in PD-L1 and ARG-1. It may be that the hypoxic signal creates a permissive condition that allows the lactate signal to be enhanced. We do not exclude the influence of hypoxia pathway on macrophage phenotype. According to the results, the hypoxia pathway and lactate signaling need to act synergistically, and their combined effect is greater than the sum of their parts.

The β2 adrenergic receptor (β2AR) is a prototypical G protein-coupled receptor (GPCR) that can increase intracellular cyclic adenosine monophosphate (cAMP) levels and activate the protein kinase A (PKA) pathway (44). Studies have also shown that β2AR signaling induces ERK activity, playing a significant regulatory role (45). In our study, we sequenced and experimentally confirmed that norepinephrine mediates macrophage immunosuppression through activation of the ERK signaling pathway via the β-receptor.

In summary, high lactate levels induce an immunosuppressive phenotype of macrophages in MPE, and this is mediated and activated by phenylalanine, tyrosine, and norepinephrine signaling. Blocking norepinephrine signaling by β-receptor blocker prevents macrophage-mediated immunosuppression and enhances anti-tumor immunity, providing a potential therapeutic strategy for β-receptor blocker treatment of MPE (**Figures 6I**).

## Data Availability

The datasets presented in this study can be found in online repositories. The names of the repository/repositories and accession number(s) can be found below: National Genomics Data Center, https://ngdc.cncb.ac.cn/gsa-human/browse/HRA009674, BioProject: PRJCA033599, GSA-human: HRA009674.
