# Supplementary material for: β-receptor blocker enhances anti-tumor immunity via inhibiting lactate-induced norepinephrine metabolism of macrophages during malignant pleural effusion
Source: Front Immunol. 2025 Jan 3;15:1497468. doi: 10.3389/fimmu.2024.1497468 (PMC11739086; doi:10.3389/fimmu.2024.1497468)
Supplement: Supplementary file 1 [file DataSheet1.docx]

**Supplementary Information**

Table S1. qPCR primers

| GENE | Forward (5' to 3') | Reverse (5' to 3') | Species |
| --- | --- | --- | --- |
| ACTB | ACAGAGCCTCGCCTTTGC | GATATCATCATCCATGGTGAGCTGG | Human |
| CD274 | CATCTTATTATGCCTTGGTGTAGCA | GGATTACGTCTCCTCCAAATGTG | Human |
| ARG1 | GTCTGTGGGAAAAGCAAGCG | CACCAGGCTGATTCTTCCGT | Human |
| PAH | GCAGACTTGCACTGGTTTCC | GCTCATGGCAGATGTCAGGT | Human |
| CD163 | TGTGGCCTGCATAGAGAGTG | TTCCCCAAAATGAGCAGAAC | Human |
| IL6 | ACTCACCTCTTCAGAACGAATTG | CCATCTTTGGAAGGTTCAGGTTG | Human |
| VEGFA | GCCTTGCCTTGCTGCTCTAC | TGATTCTGCCCTCCTCCTTCTG | Human |
| DBH | CACACCTGACTGGGAGAAAGG | CACGACCTTCTTCAACATGCGG | Human |
| DDC | TTCTGCCATGTGGGTGAA | TGCGGATATAAGCCTGCA | Human |

qPCR, quantitative polymerase chain reaction; ARG1, arginase 1; PAH, phenylalanine hydroxylase; IL, interleukin; VEGFA, vascular endothelial growth factor A; DBH, dopamine beta-hydroxylase; DDC, dopa decarboxylase.

**
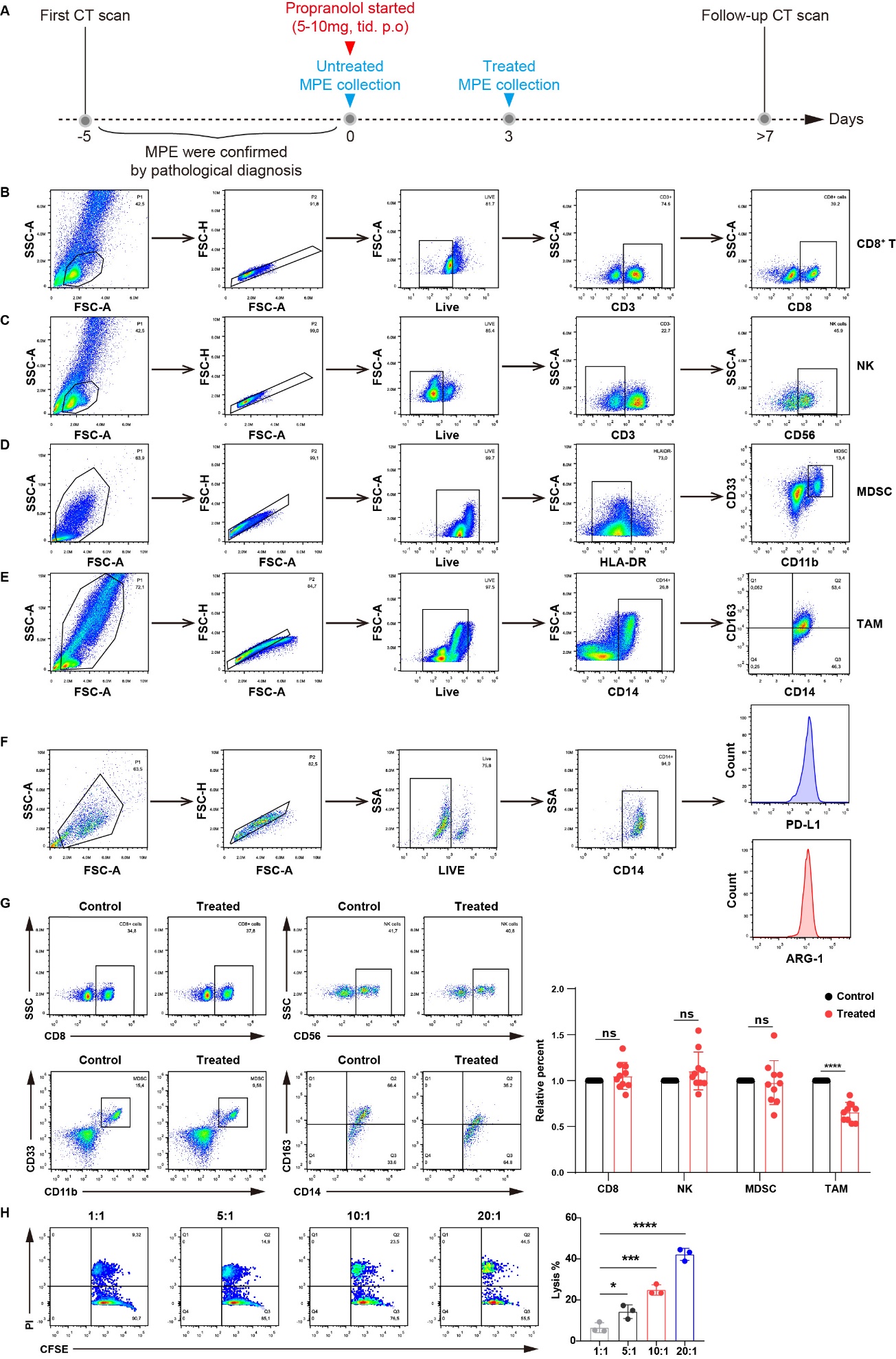
**

**Figure S1. Propranolol treatment reduces TAM frequency and enhanced anti-tumor immunity.**

(A) The timeline of diagnosis and treatment in patients received propranolol (5-10 mg/dose/time, three times/day, continuous oral administration) for heart disease and had MPE. The gating strategy for flow cytometry of the percentages of immune cells including CD8^+^ T (B), NK (C), MDSC (D), and TAM (E) cells in MPE. (F) The gating strategy for flow cytometry of the expression of PD-L1 and ARG-1 in TAMs derived from MPE. **(**G)The immune cell frequency changes in MPE treated with DMSO (control)/propranolol (10 µM) *ex vivo*. MPE samples derived from lung cancer patients (n=10) were treated with propranolol *ex vivo*. Representative image and statistical graph of flow cytometry showing changes in the frequency of immune cells including CD8^+^ T, NK, MDSC, and TAM cells. **** = P < 0.0001, ns = non-significance. (H) The effect of TAMs on NK lysis. Representative image and statistical graph of flow cytometry showing TAM-affected NK cell-induced apoptosis of tumor cells in different ratios of effector/target. * = P < 0.05, *** = P < 0.001, **** = P < 0.0001.

**
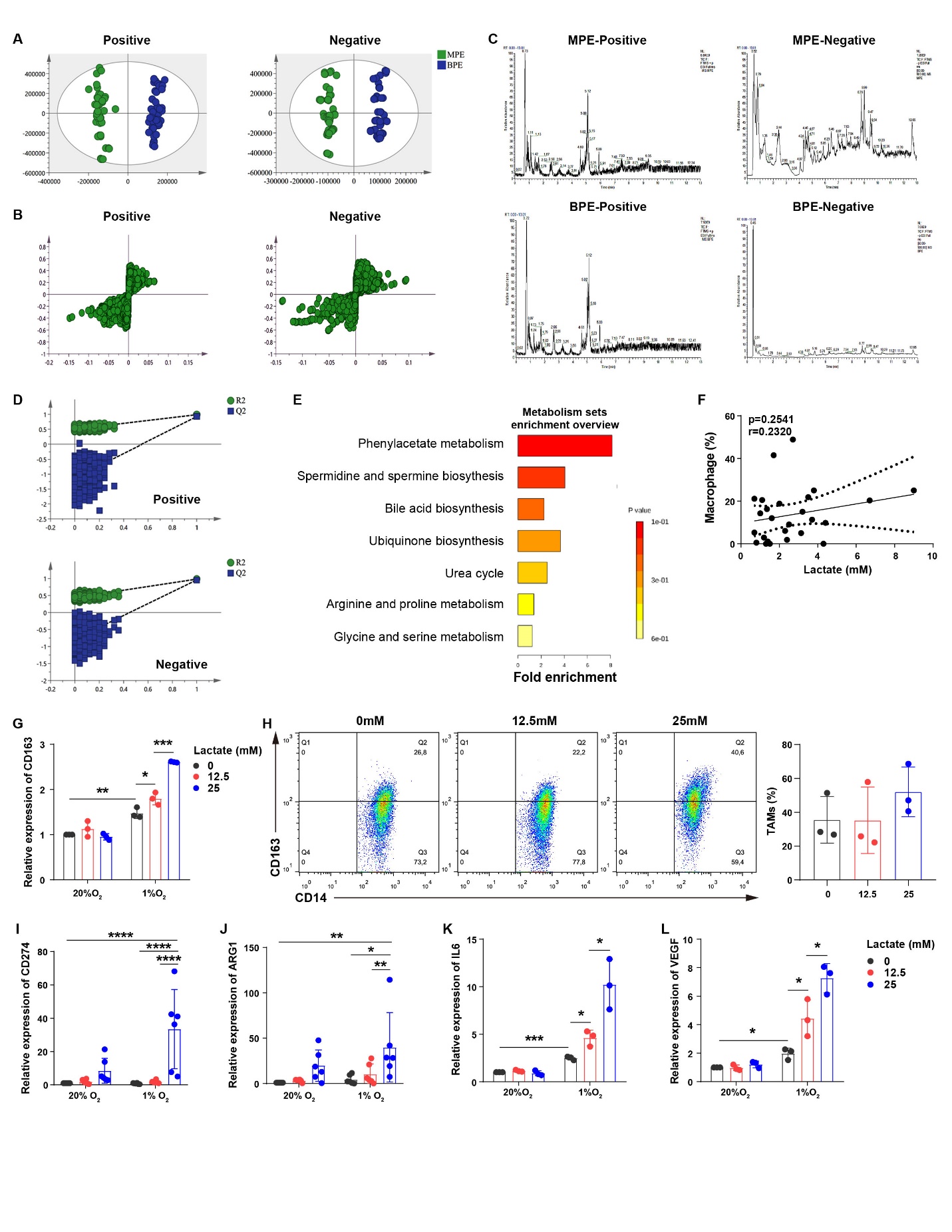
**

**Figure S2. The effect of lactate on macrophage immunosuppression.**

(A) Orthogonal partial least squares discrimination analysis (OPLS-DA) of MPE and BPE. (B) S-Plot analysis of MPE and BPE. (C) Total ion chromatogram (TIC) of MPE and BPE. (D) Permutation test of MPE and BPE. (E) Bar chart showing the different metabolism sets in MPE and BPE. (F) The correlation between CD163^+^ macrophage frequency and lactate levels in BPE. A stepwise conditional logistic regression model was used for multivariate regression analysis. (G)The mRNA expression of CD163 in lactate-treated macrophages (0, 12.5, and 25 mM) under normoxia or hypoxia was analyzed using qPCR. (H) The percentages of TAMs induced by lactate (0, 12.5, and 25 mM) under normoxia were analyzed using flow cytometry. The mRNA expression of PD-L1 (CD274) (I) and ARG-1 (J) in lactate-treated macrophages (0, 12.5, and 25 mM) under normoxia or hypoxia was analyzed using qPCR. The mRNA expression of IL-6 (K) and VEGF (L) in lactate-treated macrophages (0, 12.5, and 25 mM) under normoxia or hypoxia was analyzed using qPCR. Graph data are displayed as the mean ± SD. *p < 0.05, **p < 0.01, ***p<0.001, ****p < 0.0001.

**
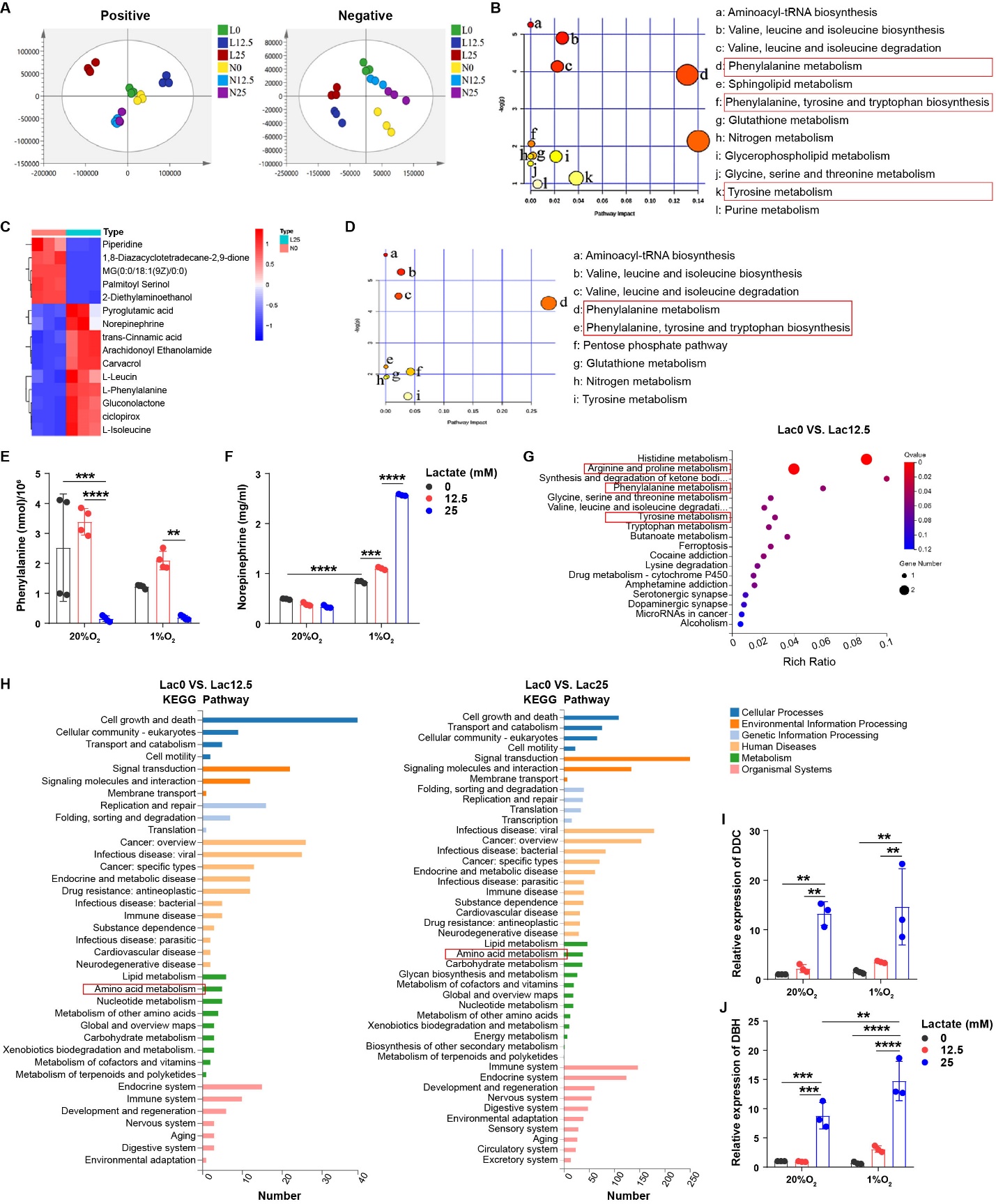
**

**Figure S3. The changes in signaling pathways involved in rhCXCL8-treated or tumor tissue-derived macrophages.**

(A) The positive and negative models of OPLS-DA of lactate-treated macrophages (0, 12.5, and 25 mM). (B) Bubble chart showing the cellular metabolism in macrophages before and after treatment with lactate (25 mM) under hypoxia. Heat map (C) and bubble chart (D) showing the cellular metabolism in macrophages after treatment with lactate (25 mM) under hypoxia and macrophages without lactate treatment under normoxia. (E**)** The levels of phenylalanine produced by lactate-treated macrophages (0, 12.5, and 25 mM) under normoxia or hypoxia were analyzed using ELISA. (F**)**The levels of norepinephrine produced by lactate-treated macrophages (0, 12.5, and 25 mM) under normoxia or hypoxia were analyzed using mass spectrometry. (G**)** Bubble chart showing the RNA-Seq results in macrophages before and after treatment with lactate (12.5 mM) under hypoxia. (H**)**KEGG pathway analysis of macrophages before and after treatment with lactate (12.5 and 25 mM) under hypoxia. The mRNA expression of DDC (I) and DBH (J) in lactate-treated macrophages (0, 12.5, and 25 mM) under normoxia or hypoxia was analyzed using qPCR. Graph data are displayed as the mean ± SD. ** = P < 0.01, *** = P < 0.001, **** = P < 0.0001.

**
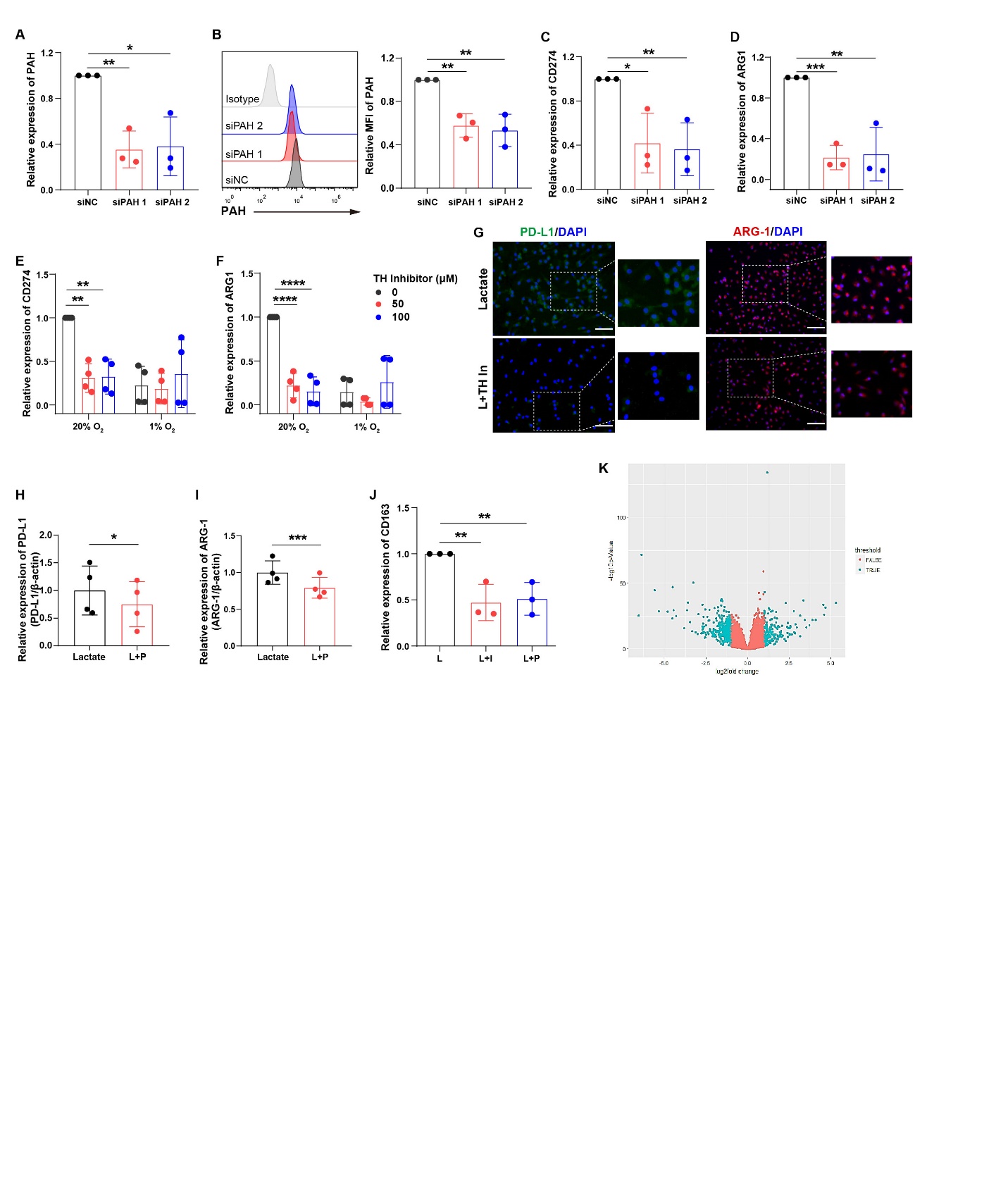
**

**Figure S4. GSEA analysis of the enriched metabolism in induced M2-like macrophages.**

(A) The mRNA expression of PAH in lactate-treated macrophages before and after transfection with siRNA-PAH under hypoxia was analyzed using qPCR. (B) The protein expression of PAH in lactate-treated macrophages before and after transfection with siRNA-PAH under hypoxia was analyzed using flow cytometry. MFI, median fluorescence intensity. The mRNA expression of PD-L1 (CD274) (C) and ARG-1 (D) in lactate-treated macrophages before and after transfection with siPAH under hypoxia was analyzed using qPCR. The mRNA expression of PD-L1 (CD274) (E) and ARG-1 (F) in lactate-treated macrophages under normoxia or hypoxia before and after treatment with TH inhibitor (50 μM) was analyzed using qPCR. (G**)** The expression of PD-L1 and ARG-1 in lactate-treated macrophages under hypoxia before and after treatment with a TH inhibitor (50 µM) was analyzed using immunofluorescence. The statistical results of PD-L1 (H) and ARG1 (I) relative expression using western blotting from Figure 4K. (J**)**The mRNA expression of CD163 in lactate-treated macrophages (L, 12.5 mM) following treatment of propranolol (P, 10 µM) or ICI18551 (I, 10 mg/mL) was analyzed using qPCR. (K**)** The different genes in lung cancer patients with high and low levels of LDH from TCGA dataset. * = *P* < 0.05, ** = *P* < 0.01, *** = *P* < 0.001, **** = *P* < 0.0001.

**
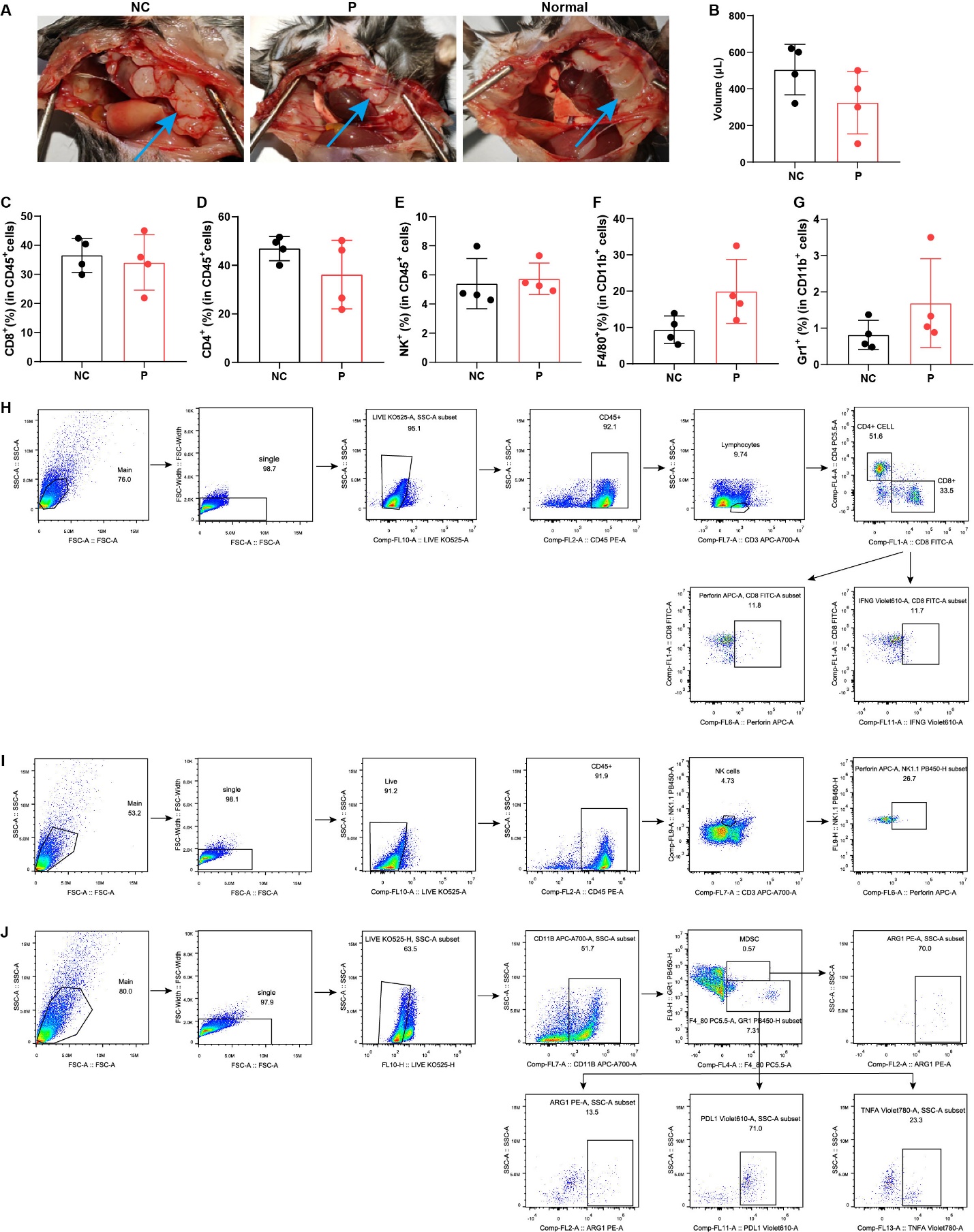
**

**Figure S5. Propranolol treatment inhibits MPE development.**

(A) Tumor burden of chest imaging from MPE mice with or without propranolol treatment. (B) Statistical result of MPE volume in mice administered with or without propranolol treatment (n = 4 mice). Flow cytometry analysis of immune cell frequency change in MPE after propranolol treatment. Percentage of CD8+ T (C), CD4+ T (D), NK (E), F4/80+ macrophage (F), and Gr1+ MDSC (G) cells in MPE with or without propranolol treatment. The gating strategy for flow cytometry of the percentages of immune cells including perforin^+^ and IFN-γ^+^ CD8^+^ T (H), perforin^+^ NK (I), ARG1^+^ MDSC and ARG1^+^, PD-L1^+^, and TNF-α^+^ TAM (J) cells in MPE model.
